# Female non-smokers’ environmental tobacco smoking exposure by public transportation mode

**DOI:** 10.1186/s40557-018-0239-7

**Published:** 2018-04-19

**Authors:** Seyoung Kim, Jin-Soo Park, Minkyu Park, Yeji Kim, Sinye Lim, Hye-Eun Lee

**Affiliations:** 1 Department of Occupational and Environmental Medicine, Kyung Hee University Hospital, Seoul, South Korea; 2 Department of Occupational and Environmental Medicine, School of Medicine Kyung Hee University, Seoul, South Korea

**Keywords:** Environmental tobacco smoking, Public transportation, Cotinine, Korean national environmental health survey

## Abstract

**Background:**

This study aimed to analyze environmental tobacco smoking exposure in female nonsmokers by public transportation mode using representative data of Koreans.

**Methods:**

Data from the Second Korean National Environmental Health Survey (2012–2014) were analyzed. Urine cotinine was analyzed by public transport behavior, secondhand smoke exposure, socioeconomic factors, and health-related factors. Participants were 1322 adult females; those with the top 75% urine cotinine concentrations were assigned to the high exposure group. A logistic regression analysis was performed considering appropriate weights and stratification according to the sample design of the Second Korean National Environmental Health Survey.

**Results:**

The geometric mean of urine cotinine concentrations differed according to public transportation modes: subway (1.66 μg/g creatinine) bus (1.77 μg/g creatinine), and taxi (1.94 μg/g creatinine). The odds ratio [OR] was calculated for the high exposure group. The OR of the taxi (2.39; 95% confidence interval, 1.00–5.69) was statistically significantly higher than the subway value (reference), and marginally significant after adjusted with life style, sociodemographic factors and involuntary smoking frequency (2.42, 95% confidence interval, 0.97–6.04).

**Conclusions:**

The odds ratio of passengers who mainly used taxis was marginally significantly higher than those of passengers who used subways and buses after adjusted with life style and sociodemographic factors. Implementation of supplementary measures and further studies on exposure to environmental tobacco smoking in taxis are warranted.

## Background

Environmental tobacco smoking (ETS) includes a mixture of > 4000 substances that are associated with cardiovascular disease in adults and inhibited respiratory system development, chronic otitis media, asthma exacerbation, upper respiratory tract irritation, and decreased intelligence quotient scores in children [1–3]. ETS is an International Agency for Research on Cancer group 1 carcinogen in human lung cancer and a suspected carcinogen for human laryngeal cancer [1]. Furthermore, a study found that ETS contributed to 1% of worldwide deaths and 0.7% of burden of disease (in disability-adjusted life years) [4].

ETS exposure frequently occurs in the general population. In fact, 40% of all children, 35% of female non-smokers, and 33% of male non-smokers are exposed worldwide [4]. Similar trends were observed in a Korean study. In the 2005 Korea National Health and Nutrition Examination, an estimated 38.6% of male and 37.4% of female non-smokers were exposed to secondhand smoke [5]. According to National Cancer Center data, the work site indoor exposure to secondhand smoke was 58.5% in males and 39.6% in females, while the home exposure to secondhand smoke was 33.8% in teenagers [6].

However, the health effects of ETS are known to be greater in female than male. According to California Environmental Protection Agency, exposure to the ETS during pregnancy negatively affects fetal growth, with elevated risks of low birth weight or “small for gestational age”. And ETS exposure increases breast cancer risk in premenopausal women [7]. In addition, female non-smokers’ lifetime incidence of lung cancer is 2.5 times greater than male [8]. The overall global statistics estimate that 53% in women are not attributable to smoking while 15% of lung cancers in men [9]. The difference is more obvious in Korea. Among 8788 Korean patients diagnosed lung cancer in 2005, never smoker was 79.7% in females and 12.7% in males [10]. Especially in Asian countries, these trends are presumed to be caused by ETS, cooking oils and fossil fuels [11]. Therefore, this study focused on female non-smokers ETS exposure.

Biomarkers that can be used for ETS exposure assessment include nicotine and its metabolites cotinine, carbon monoxide, thiocyanate, benzo[a]pyrene, and 4-(methylnitrosamino)-1-(3-pyridyl)-1-butanol. Cotinine has a relatively high sensitivity and specificity compared to the other biomarkers and is less invasive since it is measurable in urine and saliva as well as serum. In addition, its half-life is about 16.8 h, reflecting the most recent 3–4 days, making it widely applicable in various studies [12].

Most studies on ETS exposure have focused on sex, socioeconomic status, and age, but research on exposure sites other than the workplace and home is difficult to find. In a Chinese study, an analysis of a population aged 35–74 years showed that men are more commonly exposed to ETS at work and women are more commonly exposed to ETS at home [13]. Similarly, in the analysis of the ETS exposure of Koreans, men at work and women at home are at higher risk of exposure [5]. In addition, the risk of a person with a lower education level was higher than that of a person with a higher education level, and the risk of sales, service, and production workers were higher than those of professionals [5].

Many citizens use public transportation daily. According to the Public Transportation Survey Report published by the Korea Transportation Safety Authority in 2013, the number of people using public transportation (including buses and urban railways but excluding taxis and trains) nationwide with transportation cards is 13 million on weekdays and 9.3 million on weekends [14]. However, studies on the relationship between public transport and ETS exposure are rare. Until now, most studies have focused on air pollutants, including studies on the exposure of bus-commuting children to polyaromatic hydrocarbons (PAHs) and black carbon, exposure of urban workers to PAHs by commuting method, and exposure of bus and taxi drivers to volatile organic compounds (VOCs) [15–17]. Among the studies on the ETS by transportation method, there have been several studies on automobile cars. Another study demonstrated that the nicotine concentration in a vehicle increases by 1.96 times per cigarette when one smokes while driving, and another showed that internal contamination due to smoking cannot be completely prevented despite an open window [18, 19]. In addition, a study found that the mean concentration of residual nicotine of the vehicle’s surface, dust, and air is significantly higher in the vehicles of smokers with a smoking ban than in the vehicles of non-smokers, which indicates tertiary hand smoking exposure [20]. These studies suggest that public citizens can be exposed to ETS by smokers using public transportation. In addition, no study has examined the differences in exposure by public transportation mode. Therefore, this study evaluated female non-smokers ETS exposure according to modes of public transportation.

## Methods

### Study participants

This study was based on the second Korean National Environmental Health Survey (KoNEHS, 2012–2014). The second KoNEHS was designed to identify the exposure levels of the Korean population to environmentally related harmful factors and identify factors that may affect them as well as investigate the temporal and spatial distribution of environmentally related health conditions. The KoNEHS is conducted by the National Institute of Environmental Research under the Ministry of Environment every 3 years in accordance with Article 14 of the Environmental Health Law [21, 22].

The second KoNEHS was conducted in 16 cities and provinces of 400 counties. The National Population and Housing Census 2010 of Statistics Korea as the population was initially stratified into the local administrative district and the coastal area, while the secondary stratification was classified by socioeconomic level, ratio of agriculture and fishery occupation to others. A multistage stratified cluster sampling in each region was applied with the proportional allocation of the square root of the population [22].

A questionnaire survey was conducted on a total of 6478 adults aged ≥19 years with about 15 respondents per sample enumeration district. The survey consisted of 142 environmental exposure–related questionnaires, 19 clinical tests, and 21 analyses of environmental harmful substances in blood and urine specimens [22].

For the analysis, among the total of 6478 participants, we excluded 1161 smokers, 2592 subjects who answered do not use public transportation or railroad and types other than bus, taxi, and subway as their main public transportation mode. Those who answered railroads and others had fewer personnel and were excluded from the study. Also, 760 males, 367 subjects whose urinary creatinine concentration exceeded the appropriate range (0.3–3.0 g/L), and 16 subjects for whom urinary cotinine concentration data were missing and 184 subjects whose urine cotinine level was less than the method detection limit, 42 subjects who is considered as a smoker(urine cotinine > 100 μg/L), 34 outliers(< 25th percentile - 1.5*interquartile range or > 75th percentile + 1.5* interquartile range) are excluded [23–25]. The final dataset included 1322 female subjects. And this study only used the published data of the Second KoNEHS.

### Variables

#### Urinary cotinine

Urinary cotinine concentrations were analyzed by gas chromatography–mass spectrometry using spot urine. The urine cotinine was extracted with chloroform and analyzed. The sample’s concentration was read using a calibration curve constructed by the Standard Addition Method. The results of the calibration standard for assaying the calibration curves were set at a reference value of ±15%, while the precision was measured using a quality control (QC) sample and the accuracy was ensured using a calibration standard solution. The precision within the batch shall be within 15% of the relative standard deviation of the QC standard solution and the accuracy between batches within 20% of the relative standard deviation of the measurement value of the QC standard solution. The Method Detection Limit (MDL) of urinary cotinine was 0.3 μg/L. The final urinary cotinine concentration was calculated after the adjustment of the urinary creatinine concentration [22, 23].

#### Factors related to public transportation and smoking

In this study, we classified the most popular modes of public transportation (bus, taxi, subway) to investigate the relationship between ETS exposure public transportation mode. The weekly public transportation use frequency was classified as 1–6 times/7–13 times/≥14 times.

Those who answered “I have never smoked” and “I used to smoke in the past but not anymore” were included in the study as non-smokers, while those who answered “I smoke now” were classified as smokers and excluded from the study. In addition, the number of recognized secondhand smoke exposures per week was classified as None/1–2/3–4/5–6/daily.

#### Potential confounders

The sociodemographic variables and health behavior-related variables of the study participants were classified as follows. Age was divided into 10-year units from age 19 years. Household income level was divided into upper/upper-middle/lower-middle/lower according to the questionnaire. A body mass index (BMI) < 18.5/18.5–25/≥25 kg/m^2^ were classified as underweight/normal/obesity, respectively, while education level was classified as below middle school/high school graduate/college graduate. Marital status was divided into married and unmarried (single/divorce/widowed/separated). Alcohol consumption was classified as non-drinkers for participants who answered “do not drink at all” or “drank in the past but not anymore,” light drinker for drinking less than a heavy drinker, and heavy drinker for “drinking 3 times or more a week and drinking more than 5 glasses.” Exercise status was classified into exercise group for those who exercised ≥3 times a week for ≥20 min and sweating during exercise and into the non-exercise group for the rest of the participants. Region was classified as cities/rural area/coastal area/ heavy metals monitoring network(42 enumeration district where atmospheric heavy metals monitoring stations are installed) according to dataset of KoNEHS. Job was divided into non-manual(managers/professionals and related workers/clerks/service workers/sale workers), manual(skilled agricultural, forestry and fishery workers/craft and related trades workers/plant, machine operators and assemblers/elementary workers) and etc.(housewives/students/unemployed) according to Korean standard classification of occupations.

### Statistical analysis

This study applied the weights given in the guidelines for using raw data of the second KoNEHS. The sample weights were calculated through design weight, non-response adjustment, and population adjustment process [22].

We examined the subjects’ general characteristics and conducted a chi-square test to investigate the differences in the distribution of independent variables. Urinary cotinine concentrations were not normally distributed, so the values were converted to a natural logarithm to obtain the geometric mean and corrected for urinary creatinine concentration. Analysis of variance was applied to compare the geometric mean of urinary cotinine according to the public transportation modes and weekly use frequency of public transportation. Analysis of covariance was used to compare the geometric means with adjustments made to each variable. To determine the urinary cotinine concentration according to public transportation mode, the top 75% (≥3.14 μg/g creatinine) was defined as the high exposure group and a logistic regression analysis was performed [26–28]. The number of recognized secondhand smoke exposures per week was adjusted in Model 1. Age, residential area, education level, marital status, alcohol consumption, public transportation use frequency, BMI, exercise status, job classification and household income were added in Model 2. The statistical analysis was performed using IBM SPSS (version 19 for Windows) and the statistical significance level was set at *p* < 0.05.

## Results

Table 1 shows the general characteristics of the study subjects (Table 1). The participants were 1322 females with a geometric mean urinary cotinine concentration of 1.75 μg/g creatinine. There was a significant difference in urinary cotinine concentration by age. At 50–59 years of age, the peak (1.92 μg/g creatinine) was formed; thereafter, it tended to decrease with age. There was also a significant difference depending on the residential area. Coastal residents (2.00 μg/g creatinine) had a higher urine cotinine concentration than city and rural residents. The number of recognized secondhand smoke exposure per week and urinary cotinine concentration had a clear tendency (*p* < 0.001). The urinary cotinine concentration of participants who answered they were not exposed to secondhand smoke was 1.62 μg/g creatinine and tended to increase as the number of secondhand smoke exposures increased. Obese people have higher urine cotinine concentration(1.84 μg/g creatinine) than normal or underweight. In addition, urine cotinine level was higher in high school graduates (1.86 μg/g creatinine), married participants (1.85 μg/g creatinine), and low household income (1.93 μg/g creatinine).

**Table 1:** Demographic distributions of the study subjects and urinary cotinine concentration(μg/g creatinine) according to general characteristics

	Category	N(%)	GM(±SE)	95% CI	*p*-value
Total					
		1322(100)	1.75(1.03)		
Age (years)					
	19–29	182(11.5)	1.35(1.08)	1.16–1.56	0.001
	30–39	233(14.7)	1.91(1.07)	1.68–2.18	
	40–49	268(16.9)	1.90(1.06)	1.68–2.15	
	50–59	348(22.0)	1.92(1.06)	1.73–2.14	
	60–69	345(21.8)	1.67(1.05)	1.51–1.85	
	≥70	206(13.0)	1.64(1.07)	1.43–1.88	
Region					
	Cities	1303(82.4)	1.82(1.03)	1.72–1.92	0.002
	Rural area	63(4.0)	1.71(1.14)	1.33–2.21	
	Coastal area	28(1.8)	2.00(1.26)	1.24–3.23	
	Heavy Metals Monitoring Network^a^	188(11.9)	1.34(1.07)	1.17–1.54	
Involuntary smoking(per week)					
	0	1273(80.5)	1.62(1.03)	1.54–1.71	< 0.001
	1~ 2	109(6.9)	1.87(1.10)	1.55–2.27	
	3~ 4	46(2.9)	2.29(1.16)	1.69–3.11	
	5~ 6	39(2.5)	2.42(1.19)	1.69–3.46	
	≥7	115(7.3)	3.08(1.10)	2.57–3.69	
BMI (kg/m^2^)					
	Underweight	55(3.5)	1.25(1.15)	0.94–1.67	0.026
	Normal	936(59.2)	1.73(1.03)	1.62–1.84	
	Obese	591(37.4)	1.84(1.04)	1.70–2.00	
Education					
	≤ Middle school	55(3.5)	1.82(1.04)	1.68–1.97	0.012
	High school	936(59.2)	1.86(1.05)	1.70–2.04	
	≥ College	591(37.4)	1.56(1.05)	1.43–1.71	
Marital status					
	Single	439(27.7)	1.52(1.05)	1.38–1.67	0.001
	Married	1143(72.3)	1.85(1.03)	1.75–1.96	
Household income					
	Low	463(29.3)	1.93(1.05)	1.76–2.13	0.003
	Mid-low	757(47.9)	1.78(1.04)	1.66–1.92	
	Mid-high	352(22.3)	1.49(1.05)	1.35–1.65	
	High	10(0.6)	1.91(1.35)	0.96–3.79	
Alcohol					
	None	818(51.7)	1.73(1.04)	1.61–1.85	0.363
	Light drinker^b^	734(46.4)	1.76(1.04)	1.64–1.90	
	Heavy drinker^c^	30(1.9)	2.25(1.22)	1.48–3.40	
Exercise					
	No	1183(74.8)	1.75(1.03)	1.66–1.86	0.986
	Yes^d^	399(25.2)	1.75(1.05)	1.58–1.94	
Job classification					
	non-manual	329(24.9)	1.88(1.05)	1.70–2.09	0.263
	manual	224(16.9)	1.72(1.07)	1.51–1.95	
	housewife, student, unemployed	769(58.2)	1.71(1.03)	1.60–1.82	

*p value calculated by chi-square tests *GM* geometric mean/*SE* standard error /*CI* confidence interval Heavy Metals Monitoring Network^a^: 42 enumeration district where atmospheric heavy metals monitoring stations are installed. Light drinker^b^: drinking less than heavy drinker/Heavy drinker^c^: drinking ≥3 times a week and ≥ 7 glasses per occasion for male (≥5 glasses for female). Exercise^d^: exercising ≥3 times a week for ≥20 min with sweating

Table 2 summarizes the changes in urinary cotinine concentrations by public transportation method and the weekly public transportation use frequency before and after adjustment for related factors such as age, BMI, residence area, the number of recognized secondhand smoke exposure per week, education level, marital status, and household income (Table 2). The relative urinary cotinine concentration according to public transportation mode was the lowest in the subway (1.66 μg/g creatinine), bus (1.77 μg/g creatinine) and taxi (1.94 μg/g creatinine) groups before adjustment. And these trends are similar in the subway (1.72 μg/g creatinine), bus (1.75 μg/g creatinine) and taxi (2.00 μg/g creatinine) groups after adjustment. But there was no statistically significant difference between the pre-adjusted (*p* = 0.401) and post-adjusted (*p* = 0.499) values. Moreover, there was no tendency or statistically significant difference in urinary cotinine concentrations between before (*p* = 0.675) and after (*p* = 0.774) adjustment.

**Table 2:** Urinary geometric mean (GM) concentration of creatinine(μg/g creatinine) categorized by public transportation usage

			Crude			Adjusted^a^		
	Category	N(%)	GM(±SE)	95% CI	*p*-value	GM(±SE)	95% CI	*p*-value
Modes of transportation								
	Subway	262(19.8)	1.66(1.06)	1.48–1.85	.401	1.72(1.06)	1.54–1.92	.499
	Bus	1002(75.8)	1.77(1.03)	1.67–1.87		1.75(1.03)	1.65–1.85	
	Taxi	58(4.4)	1.94(1.13)	1.53–2.46		2.00(1.12)	1.59–2.52	
Using frequency(per week)								
	1–6	826(62.5)	1.72(1.03)	1.62–1.83	.675	1.73(1.03)	1.63–1.84	.774
	7–13	414(31.3)	1.80(1.05)	1.65–1.97		1.80(1.05)	1.65–1.96	
	≥14	82(6.2)	1.80(1.11)	1.48–2.20		1.77(1.10)	1.46–2.15	

^a^Adjusted by Age, BMI, Education, Household income, Involuntary smoking, Marital status, Region *GM* Geometric Mean *SE* Standard Error *CI* Confidence Interval

Odds ratios (OR) were calculated by logistic regression analysis according to modes of public transportation for the top 25% urinary cotinine concentration (≥3.14 μg/g creatinine; Table 3). In the case of taxis, the OR was 2.39 (95% confidence interval [CI], 1.00–5.69) in model 1 and 2.42 (95% CI, 0.97–6.04) in model 2. The OR of the bus group was higher than that of the subway group in models 1 and 2, but the difference was not statistically significant.

**Table 3:** Odds ratios (OR) and 95% confidence intervals (CI) of public transportation modes of high urinary cotinine concentration (≥3.14 μg/g creatinine) compared to low urinary cotinine concentration

Category		OR(95% CI)
Model 1^a^		
	Subway	Reference
	Bus	1.30(0.87–1.95)
	Taxi	2.39(1.00–5.69)
Model 2^b^		
	Subway	Reference
	Bus	1.21(0.80–1.84)
	Taxi	2.42(0.97–6.04)

^a^Model 1: Adjusted with involuntary smoking frequency ^b^Model 2: Adjusted with involuntary smoking frequency, alcohol, age, body mass index, education, exercise, household income, job classification, marital status, region, using frequency of public transportation

## Discussion

A total of 1322 Korean female non-smokers were analyzed in this study, which revealed that the risk of high ETS exposure was higher in taxi users. The GM of urine cotinine by transportation modes was higher in order of subway, bus, taxi, although not statistically significant. And OR according to modes of public transportation was higher in taxi. It was marginally significant after adjusted with life style, sociodemographic factors and involuntary smoking frequency.

Research on public transport use and ETS exposure is difficult to find, but similar studies were found. Jo et al. measured the VOCs inside vehicles during working hours in Seoul and found that the mean VOC concentration in taxis was higher than that in buses [17]. In this study, the possible reasons for the difference included vehicle height, cabin volume, and indirect smoking. This finding suggests that the high urinary cotinine concentration of passengers using taxis may be related to the vehicle’s size. In other studies in Korea and China, the VOC concentration was analyzed according to transportation modes. The VOCs in taxis or private vehicles were higher than those on buses [29, 30]. Jo et al. also showed that the VOC concentration of a smoking driver’s vehicle was 1.2–1.9-fold higher than a non-smoking driver’s vehicle or non-smoking buses and taxis [17]. It can be seen from the above that even if smoking is prohibited in a vehicle, VOC exposure in a smoking driver’s vehicle is possible.

ETS includes second-hand smoking (SHS) and third-hand smoking (THS). SHS refers to a mixture of sidestream smoke and exhaled mainstream smoke from the lungs of smokers. THS refers to secondary pollutants that are generated by re-emission of residual pollutants left on the surface or dust of a smoking area after smoking, in a gaseous state, or by reaction with the environment [31]. Because smoking is banned in most public transportation modes, the importance of THS exposure is more pronounced than the actual smoking or SHS. Matt et al. analyzed the concentration of nicotine in the surface, dust, and air in > 100 used cars divided into three groups; nonsmoker, smoker with car smoking ban, and smoker without car smoking ban. The concentrations of nicotine in the surface, dust, and air were higher in the order of smoker without car smoking ban, smoker with car smoking ban, and non-smoker. In addition, the nicotine concentration of the dust (*p* = 0.259) and the surface (*p* = 0.430) of smoker without car smoking ban group were not different from smoker with car smoking ban group; on the other hand, those were 84 times higher on the surface and 3.4 times higher in the dust than in the non-smoker group [20]. In the same researcher’s study of rental cars, smoking residues accumulate inside the vehicle because of the longer the cumulative mileage, and the higher the age, the higher the surface, dust and air nicotine concentrations in the non-smoking and smoking vehicles [32]. This is because of the characteristics of THS that are accumulated repeatedly by adsorption and desorption of smoking byproducts such as nicotine on the surfaces of peripheral substances [31]. Thus, non-smokers can be affected by the smoking of drivers and passengers on public transport.

There are a few reasons why the mean cotinine concentrations and OR were high in the urine of the citizens who mainly used taxis. First, there may be an effect due to the size difference of the vehicles. Jones et al. reported that the median nicotine concentration (32.3 μg/L) in a compact size vehicle was higher than the mid-size vehicle (7.5 μg/L) when smoked while driving to a privately owned passenger vehicle [18]. Jo et al. analyzed the concentrations of VOCs in the buses and taxis during operation, which showed that the VOC concentrations in taxis were higher than those in buses. One of the reasons for this is that pollutants are being diluted because of the large cabin volume of buses [17]. Also in this study, vehicle size can be considered an important factor since the cotinine concentration is higher in the order of subway, bus, and taxi as vehicle size decreases.

Next, the difference in external ventilation between public transports can be considered. Several studies have shown that as the time and frequency of window opening increases, the concentration of contaminants inside the vehicle decreases more rapidly. According to Ott et al., the air-change per hour of pollutants (carbon dioxide, Particulate Matter 2.5) caused by smoking in the car increased by 10 times when the window was opened about 3 in. compared to when the window was closed [33]. In addition, an interaction between ventilation and smoking was observed in the previous studies. The increase in the average respirable suspended particles after smoking was greater under air conditioned ventilation than open-window ventilation [18, 34]. In this study, it was impossible to directly compare the ventilation states of subways, buses, and taxis. However, considering the characteristics of public transportation, which closes most of the windows during operation time, the external ventilation is likely correlated with the number of times the vehicle stops. In the case of subways and buses, the distance between stops is about once every 2 or 3 min, whereas the taxi stops less frequently. Also, because of the number of passengers getting on and off for subways and buses are relatively high, the door is opened for a longer period of time during each stop. Therefore, subways and buses are expected to have better ventilation conditions than taxis.

Third, the cause can be estimated from the lack of legal regulations of smoking in vehicles. Article 9 of the National Health Promotion Act prohibits smoking in vehicles with more than 16 passengers and in children’s transportation. However, taxis are exempt from this requirement since they accommodate only four passengers [35]. In accordance with Article 26 of the Passenger Transport Service Act, taxi drivers are banned from smoking in their vehicles, while taxi passengers are not [36]. In addition, since taxis only have a small number of passengers (1–4), passengers are more likely to try smoking in the car, and there is currently no way for taxi drivers to stop passengers from smoking.

The effect of the smoking ban policy is obvious. In a study that observed at changes after the smoking ban policy of the taxi in Lisbon, 76.9% of taxi drivers accepted the passenger’s smoking needs before the law versus 16.8% after the law was enacted. The most common reason for not accepting the passenger’s request was the law and the fine (71.2%) [37]. In a study of urine cotinine concentration before versus after the strengthening of smoking regulations in public places for non-smoking workers in Korea, the concentration was lower after (2011, 2.72 ng/mL) the strengthening than before (2009, 2.91 ng/mL; *p* < 0.001) [38]. There have been a few proposed smoking bans in taxi passengers in Korea, but they have not yet been passed or enacted. It is necessary to find a way to prevent passengers from smoking within taxis.

Studies of ETS exposure have shown a similar pattern. An analysis of 8270 workers in Sweden using the Scania Public Health Survey showed that ETS exposure occurred mainly in individuals who were male, of younger age, or had lower socioeconomic status [39]. In the present study, the urinary cotinine concentration was statistically significant in terms of age, residence area, education level, and alcohol consumption. The lower the socioeconomic status and age, the higher the urinary cotinine concentration. In addition, in previous studies in Korea and China, females were more commonly exposed to ETS at home than at work [5, 13]. In this study, married women had higher urinary cotinine concentrations than unmarried women. This is likely to be associated with home ETS exposure in females.

The limitations of this study are as follows. First, the questionnaire for the use of transportation asked participants “modes of public transportation mainly used.” Therefore, it does not reflect the complex use of various means such as subways, buses, and taxis. Second, other exposures than public transportation(i.e. family exposure, occupational exposure etc.) could not be excluded because the questionnaire was not divided into exposure places. Therefore, other exposures are regarded as random effect. It is necessary to modify the questionnaire in the future. Third, size of the study subjects was rather small (only 58 taxi users), thus statistical significance might not be secured. In addition, we could not figure out the smoking rate of drivers by each public transportation modes and then adjust the effect. And since the urine sample collection method is spot urine, it is necessary to collect samples by 24-h urine to ensure more precise results.

Despite these limitations, this study examined the ETS exposure of Korean adults using the second KoNEHS, which represents the general population of Korea, and found a link between public transport and ETS exposure.

## Conclusion

Compared with subway and buses, the urinary cotinine concentrations and OR of passengers who mainly used taxis was higher. Differences in vehicle size and stopping frequency are possible causes. Also, due to the current law, taxis are classified as vehicles with less than 16 passengers, which seems to be lack of grounds to prevent passengers from smoking. Supplemental measures and further studies on ETS exposure in taxis are needed.

## Abbreviations

ANCOVA: Analysis of covariance
ANOVA: Analysis of variance
BMI: Body mass index
CI: Confidence interval
ETS: Environmental tobacco smoking
IRB: Institutional Review Board
KoNEHS: Korean National Environmental Health Survey
MDL: Method detection limit
OR: Odds ratio
PAHs: Poly aromatic hydrocarbons
QC: Quality control
SHS: Second hand smoking
THS: Third hand smoking
VOCs: Volatile organic compounds
